# Dr. Henry A. Wise

**Published:** 1904-08

**Authors:** 


Dr. Henry A. Wise, of Williamsburg, Va., died at St. Luke's Hospital, Richmond, May 5, 1904, aged 30 years. He was born in Williamsburg, where he grew up to manhood and practised his profession. His preliminary education was obtained under the embarrassments of partial invalidism due to hip disease, but he completed his academic studies at William and Mary College and took his medical degree at the University College of Medicine, Richmond, in 1896. Dr. Wise began the practice of medicine in association with his father in the city of his birth where he acquired an extended clientele and was regarded as the foremost physician of his years on the Virginia peninsula. He held the office of examining surgeon at Hampton, Va., and had been president of the alumni association of his alma mater. He was a man of accomplishments, sterling integrity, professional skill and courteous manners. He sprang from one of the most distinguished families of Virginia and leaves a large group of relatives and friends in grief at his early demise.

